# Real-world treatment patterns and clinical outcomes in patients treated with eribulin after prior phosphoinositide 3-Kinase inhibitor treatment for metastatic breast cancer

**DOI:** 10.1007/s10549-023-07080-1

**Published:** 2024-02-04

**Authors:** Ravi K. Goyal, Jingchuan Zhang, Keith L. Davis, Martina Sluga-O’Callaghan, Peter A. Kaufman

**Affiliations:** 1 RTI Health Solutions, Research Triangle Park, NC USA; 2 Eisai Inc, Nutley, NJ USA; 3 Larner College of Medicine, Division of Hematology/Oncology, University of Vermont Cancer Center, 111 Colchester Avenue, EP2, Burlington, VT 05401 USA

**Keywords:** Eribulin, Real-world, Metastatic breast cancer, PI3K inhibitor, CDK4/6 inhibitors

## Abstract

**Purpose:**

In 2010, the US Food and Drug Administration approved eribulin for the treatment of metastatic breast cancer (MBC). Since then, the treatment landscape has evolved with many new therapy classes, a more recent one being the small molecule inhibitors of phosphoinositide 3 kinase (PI3K). We sought to characterize the treatment patterns and clinical outcomes of patients with MBC who received eribulin following prior treatment with a PI3K inhibitor.

**Methods:**

A retrospective cohort study based on medical record review included MBC patients who initiated eribulin between March 2019 and September 2020 following prior treatment with a PI3K inhibitor was conducted. Patient demographics, treatment characteristics, and clinical outcomes were analyzed descriptively. Real-world progression-free survival (rwPFS) and overall survival (OS) were estimated from the initiation of eribulin therapy using Kaplan-Meier analyses.

**Results:**

82 eligible patients were included. Patients’ median age at eribulin initiation was 62 years; 86.5% had hormone receptor–positive, human epidermal growth factor receptor 2–negative tumors. Eribulin was most often administered in the second or third line (82.9%) in the metastatic setting. Best overall response on eribulin was reported as complete or partial response in 72% of the patients. The median rwPFS was 18.9 months (95% confidence interval [CI], 12.4-not estimable); median OS was not reached. The estimated rwPFS and OS rates at 12 months were 63.3% (95% CI, 50.5–73.7) and 82.6% (95% CI, 72.4–89.3), respectively.

**Conclusion:**

Our real-world study suggests that eribulin may be a potential treatment option for MBC patients who fail a prior PI3K inhibitor.

## Introduction

Breast cancer is one of the most prevalent forms of cancer, surpassing lung cancer in 2020 as the most commonly diagnosed cancer worldwide [1]. In the United States (US), the National Cancer Institute estimated there would be 287,850 new cases of breast cancer in 2022, accounting for 15% of all new cancer cases [2]. Among patients with newly diagnosed breast cancer each year, approximately 6% are diagnosed at the metastatic stage. Nearly three-quarters (72%) of patients with metastatic breast cancer (MBC) were originally diagnosed with local disease [3]. While the 5-year survival rate for breast cancer is 90.6%, it drops precipitously to nearly 30.0% in those with cancer metastasized to distant sites [2].

Because of the aggressive nature of MBC, the goal of treatment is to relieve symptoms, slow disease progression, and prolong life. Treatment decisions for MBC are often made based on cancer subtype, determined by their expression of hormone receptors (HRs) or human epidermal growth factor receptor 2 (HER2) [4]. Common systemic therapies that have traditionally been used in early-line treatments for HR-positive (HR+) MBC include hormone therapies, like fulvestrant and tamoxifen, and chemotherapies, like taxanes and anthracyclines. In 2010, eribulin mesylate was approved in the US to treat MBC patients who have received at least 2 prior chemotherapy regimens for metastatic disease, including an anthracycline and a taxane in either the metastatic or adjuvant setting. The approval was based on the survival benefit of eribulin over treatment of a physician’s choice in the EMBRACE clinical trial [5].

Since the approval of eribulin, the treatment landscape for MBC has shifted significantly with the advent of new classes of treatments, including the phosphoinositide 3 kinase (PI3K) inhibitor alpelisib and cyclin-dependent kinases 4/6 inhibitors (CDK4/6i). CDK4/6i were first approved in 2015 and are the current standard of care for first-line therapy in HR+/HER2-negative (HER2–) MBC when used in combination with endocrine therapy [4, 6–9]. Mutations in the gene encoding the catalytic α-subunit of PI3K (*PIK3CA*) drive cancer proliferation and metastasis pathways and are found in approximately 30-40% of HR+/HER2– tumors [10–14]. Alpelisib, a PI3K inhibitor, was approved in 2019 in combination with fulvestrant to treat patients with HR+/HER2–, *PIK3CA*-mutated, advanced or MBC who progressed on or after endocrine therapy [10, 15, 16].

The real-world effectiveness of eribulin treatment has been thoroughly evaluated in patients with MBC in US clinical practice [17, 18]. However, given the recent approval of alpelisib, there are no data available on the treatment patterns and clinical outcomes associated with eribulin in patients previously treated with alpelisib. In view of ongoing therapeutic advances in MBC and the paucity of real-world evidence on the clinical effectiveness of eribulin post–PI3K inhibitor therapy, in this study, we assessed patient characteristics, treatment patterns, and clinical outcomes among patients with MBC who initiated eribulin after treatment with a PI3K inhibitor in the US. Additionally, we assessed these study measures and outcomes in a subgroup of patients who received both a PI3K inhibitor and a CDK4/6i prior to initiating eribulin.

## Methods

### Study design

A retrospective, noninterventional review of medical record data of MBC patients treated with eribulin post receiving a PI3K inhibitor was conducted. Physicians consenting to participate in the study were recruited from academic and community practices across all regions of the US and provided deidentified data from patient medical records. Participating oncologists were required to have been in practice for at least 2 years managing patients with MBC and to have been responsible for treatment decisions regarding these patients. Patients included in this study were female and 18 years of age or older at eribulin initiation, with a histologically confirmed diagnosis of MBC, who had initiated treatment with eribulin between March 1, 2019, and September 30, 2020, after a prior line of therapy containing alpelisib in either clinical practice or in a clinical trial setting. Patients were excluded if they received eribulin as part of a clinical trial during the study period or had evidence of other malignant neoplasms (except nonmelanoma skin cancer and early-stage breast cancer) prior to diagnosis of MBC. Because of the use of deidentified patient data, the Institutional Review Board (IRB) at the Research Triangle Institute determined that this study did not require full IRB review or informed consent from patients.

### Study measures and outcomes

Patient demographics, clinical characteristics, and eribulin treatment characteristics were collected by physicians from the information available in patient medical records.

Clinical outcomes from the initiation of eribulin treatment were assessed. The physician-reported real-world best overall response was abstracted from patient’s medical chart as recorded by the physician at the time of assessment and categorized as complete response (CR), partial response (PR), stable disease (SD), or progressive disease (PD). Real-world progression-free survival (rwPFS) was calculated from the date of eribulin initiation to the earliest date of physician-reported progression or death due to any cause while on eribulin treatment or within 90 days after eribulin treatment discontinuation, as long as no subsequent treatments had been initiated. Patients with no documented progression or death event were censored at 90 days after eribulin discontinuation, start of the new line of therapy, or at the last available follow-up in the medical record, whichever was earliest. Overall survival (OS) was calculated from the date of eribulin initiation to death due to any cause; if no death event occurred, patients were censored at the last available follow-up in the medical record.

### Statistical analyses

All analyses were conducted using SAS Studio (SAS Institute, Inc.; 2011). Mean, median, interquartile range, and standard deviation were reported for continuous variables. Categorical values are provided as counts and frequencies. Kaplan-Meier analyses were performed to assess time to event variables, such as time to treatment discontinuation, rwPFS, and OS. All study measures and clinical outcomes were analyzed among all eribulin-treated patients who previously received a PI3K inhibitor therapy. Additionally, as a subgroup assessment, the outcomes of patients treated with both a PI3K inhibitor and a CDK4/6i prior to eribulin initiation (referred to as “post PI3K inhibitor and CDK4/6i subgroup”) were analyzed separately.

## Results

### Physician characteristics

A total of 36 physicians participated in this study. Almost equal proportion of physicians were associated with academic practice (47%) and community practice (50%). Likewise, similar distributions were seen for practice size, with 50% of physicians practicing at large practices (≥ 10 oncologists) and 47% practicing at small or intermediate practices (2–9 oncologists); only 3% practiced individually at a solo facility. The median years spent in practice was 17 years (range, 6–30), and on average, the physicians spent over 90% of their time in direct patient care. Physician offices were located throughout the US (25.0% in the Northeast, 25.0% in the South, 22.2% in the Midwest, and 27.8% in the West) and distributed in urban (63.9%) and suburban (36.1%) settings.

### Patient characteristics

A total of 82 patients with MBC who received eribulin after receiving a prior PI3K inhibitor were included in our study; a subset of 35 patients received both a PI3K inhibitor and a CDK4/6i prior to eribulin initiation. The median age of all patients was 62 years at eribulin initiation; 70.7% of the patients were White/Caucasian, 23.2% were African Americans, and 14.6% of all patients were of Hispanic ethnicity. Among patients who received both alpelisib and a CDK4/6i prior to eribulin, the median age at eribulin initiation was 66 years, 68.6% were White/Caucasian, 25.7% were African Americans, and 11.4% were Hispanic. The median duration of follow-up from the initiation of eribulin treatment for all patients was 12.2 months. At the end of follow-up, the majority (73.2%) of patients were alive. Full demographic and clinical characteristics can be found in Table 1.

**Table 1:** Demographic and Clinical Characteristics at Baseline

	All patients with prior PI3K inhibitor treatment (n = 82)	Patients with prior PI3K inhibitor and CDK4/6i treatment (n = 35)
**Age at eribulin initiation, years**		
Mean (SD)	63.2 (9.4)	65.5 (9.0)
Median	62.2	66.1
**Race, n (%)**		
White	58 (70.7)	24 (68.6)
Black/African American	19 (23.2)	9 (25.7)
Asian	4 (4.9)	2 (5.7)
Native Hawaiian or other Pacific Islander	1 (1.2)	0 (0)
**Ethnicity, n (%)**		
Hispanic/Latino	12 (14.6)	4 (11.4)
Not Hispanic/Latino	66 (80.5)	31 (88.6)
Unknown	4 (4.9)	0 (0)
**Geographic region of residence, n (%)**		
Northeast	19 (23.2)	10 (28.6)
South	24 (29.3)	8 (22.9)
Midwest	13 (15.9)	4 (11.4)
West	25 (30.5)	12 (34.3)
Unknown	1 (1.2)	1 (2.8)
**Biomarker status, n (%)**		
HR+/HER2–	69 (84.2)	34 (97.1)
HR+/HER2+	2 (2.4)	1 (2.9)
HR–/HER2+	3 (3.7)	0 (0)
Triple negative	8 (9.8)	0 (0)
**Metastatic sites at eribulin initiation, n (%)**		
Bone	44 (53.7)	20 (57.1)
Brain	6 (7.3)	1 (2.9)
Liver	29 (35.4)	16 (45.7)
Lung	29 (35.4)	19 (54.3)
Lymph nodes	37 (45.1)	13 (37.1)
**ECOG performance status at eribulin initiation, n (%)**		
0 (Asymptomatic)	5 (6.1)	2 (5.7)
1 (Symptomatic, completely ambulatory)	59 (72.0)	23 (65.7)
2 (Symptomatic, < 50% in bed during the day)	9 (11.0)	5 (14.3)
Unknown	9 (11.0)	5 (14.3)
**Charlson Comorbidity Index score**		
Mean (SD)	1.6 (1.6)	1.3 (0.9)
**Vital status at the end of follow-up, n (%)**		
Alive	60 (73.2)	22 (62.9)
Dead	21 (25.6)	13 (37.1)
Unknown	1 (1.2)	0 (0)
**Number of prior therapy lines involving chemotherapy**^**a**^, **n (%)**		
0	68 (82.9)	28 (80.0)
1	8 (9.8)	5 (14.3)
≥ 2	6 (7.3)	2 (5.7)
**Number of prior therapy lines involving hormonal therapy**^**a**^, **n (%)**		
0	62 (75.6)	22 (62.9)
1	14 (17.1)	10 (28.6)
2	6 (7.3)	3 (8.6)

CDK4/6i = cyclin-dependent kinases 4/6 inhibitors; ECOG = Eastern Cooperative Oncology Group; HER2 = human epidermal growth factor receptor 2; HER2– = HER2 negative; HER2 + = HER2 positive; HR = hormone receptor; PI3K = phosphoinositide 3 kinase; SD = standard deviation

^a^ Monotherapy or combination therapies received before eribulin treatment in the metastatic setting

Based on the available medical history data, any early-stage breast cancer diagnosis prior to MBC was reported for 12.2% of patients. The most common MBC subtype was HR+/HER2– disease (84.2% of all patients; 97.1% of patients in the post PI3K inhibitor and CDK4/6i subgroup); 9.8% of patients in the overall population had triple-negative disease, and 6.1% had HER2-positive (HER2+) disease. Mutations in *PIK3CA* were commonly reported (69.5%). The most common metastatic sites involved at the time of eribulin initiation were bone (53.7%), lymph nodes (45.1%), liver (35.4%), and lung (35.4%). The Eastern Cooperative Oncology Group (ECOG) performance status at eribulin treatment initiation was reported as 0 or 1 for 78.0% of all patients and 71.4% of patients in the post PI3K inhibitor and CDK4/6i subgroup. The mean Charlson Comorbidity Index score measured at MBC diagnosis was 1.6 (standard deviation, 1.6), and the most commonly reported comorbidities were hypertension (45.1%), followed by depression (24.4%), diabetes (18.3%), and chronic pulmonary disease (13.4%).

### Treatment patterns

Patients received between 2 and 6 lines of systemic therapy after MBC diagnosis. The median time to initiation of first-line therapy since MBC diagnosis was 0.4 months (Table 2). Eribulin use was reported as second-line therapy (45.1%), third-line therapy (37.8%), and fourth-line therapy or later (17.1%) in the metastatic setting in the overall study population. In patients who received both a prior PI3K inhibitor and a prior CDK4/6i, most (62.9%) received eribulin in the third line, 17.1% received it in the fourth line, and 20.0% received it in the fifth line in the metastatic setting (Table 2).

**Table 2:** Eribulin Treatment Characteristics

	All patients with prior PI3K inhibitor treatment (n = 82)	Patients with prior PI3K inhibitor and CDK4/6i treatment (n = 35)
**Distribution of therapy line in the metastatic setting in which eribulin was initiated, n (%)**		
Second line	37 (45.1)	0 (0)
Third line	31 (37.8)	22 (62.9)
Fourth line	6 (7.3)	6 (17.1)
Fifth line	8 (9.8)	7 (20.0)
**Eribulin therapy status at the time of data collection (end of follow-up), n (%)**		
Discontinued	51 (62.2)	20 (57.1)
Ongoing	31 (37.8)	15 (42.9)
**Duration of eribulin therapy, months**		
Among all patients		
Mean (SD)	9.9 (6.0)	9.4 (5.0)
Median	9.5	8.7
Among patients who discontinued therapy		
Mean (SD)	7.1 (4.3)	6.3 (2.8)
Median	6.1	5.6
Among patients whose therapy was ongoing at end of follow-up		
Mean (SD)	14.6 (5.6)	13.6 (4.0)
Median	13.2	13.2
**Time to subsequent therapy line**,^**a**^**months**		
Among patients who received subsequent therapy line, n (%)	15 (18.3)	6 (17.1)
Mean (SD)	0.7 (0.7)	0.5 (0.1)
Median	0.5	0.4

CDK4/6i = cyclin-dependent kinases 4/6 inhibitors; PI3K = phosphoinositide 3 kinase; SD = standard deviation

^a^Time to initiation of subsequent therapy line was assessed from the end of eribulin therapy

In the overall study population, alpelisib (PI3K inhibitor) treatment was the most frequent first-line treatment regimen (41.5%), followed by CDK4/6i and endocrine therapy combinations (common regimens included palbociclib + letrozole, 17.1%; abemaciclib + fulvestrant, 8.5%; and palbociclib + fulvestrant, 8.5%). In the subgroup of patients who had used both a PI3K inhibitor and a CDK4/6i in therapy lines prior to eribulin initiation, CDK4/6i therapy was always administered prior to the treatment line with PI3K inhibitor alpelisib. In the overall patient cohort, 82.9% of patients did not receive any prior chemotherapy in the metastatic setting before initiating eribulin treatment, 9.8% received 1 prior line of chemotherapy, and 7.3% received at least 2 lines of chemotherapy; 75.6% of patients did not receive prior hormonal therapy in the metastatic setting, and 24.4% of patients received 1–2 prior hormonal therapies. In patients who received both a prior PI3K inhibitor and a prior CDK4/6i, 80.0% of patients did not receive any prior chemotherapy and 20.0% received 1–2 prior lines of chemotherapy; 62.9% of the patients did not receive prior hormonal therapy, and 37.1% received 1–2 prior hormonal therapies.

At last follow-up, eribulin treatment was ongoing for 37.8% of patients (Table 2). The median eribulin treatment duration was 6.1 months among those who had discontinued eribulin and 13.2 months among those who were still on treatment. The estimated median time to eribulin treatment discontinuation based on Kaplan-Meier analyses was 10.7 months (95% confidence interval [CI], 7.3–13.3). Among the 62.2% of patients who discontinued eribulin at last follow-up, the most common reasons for discontinuation were PD (52.9%) and patient decision (29.4%).

### Clinical outcomes

**Fig. 1:**
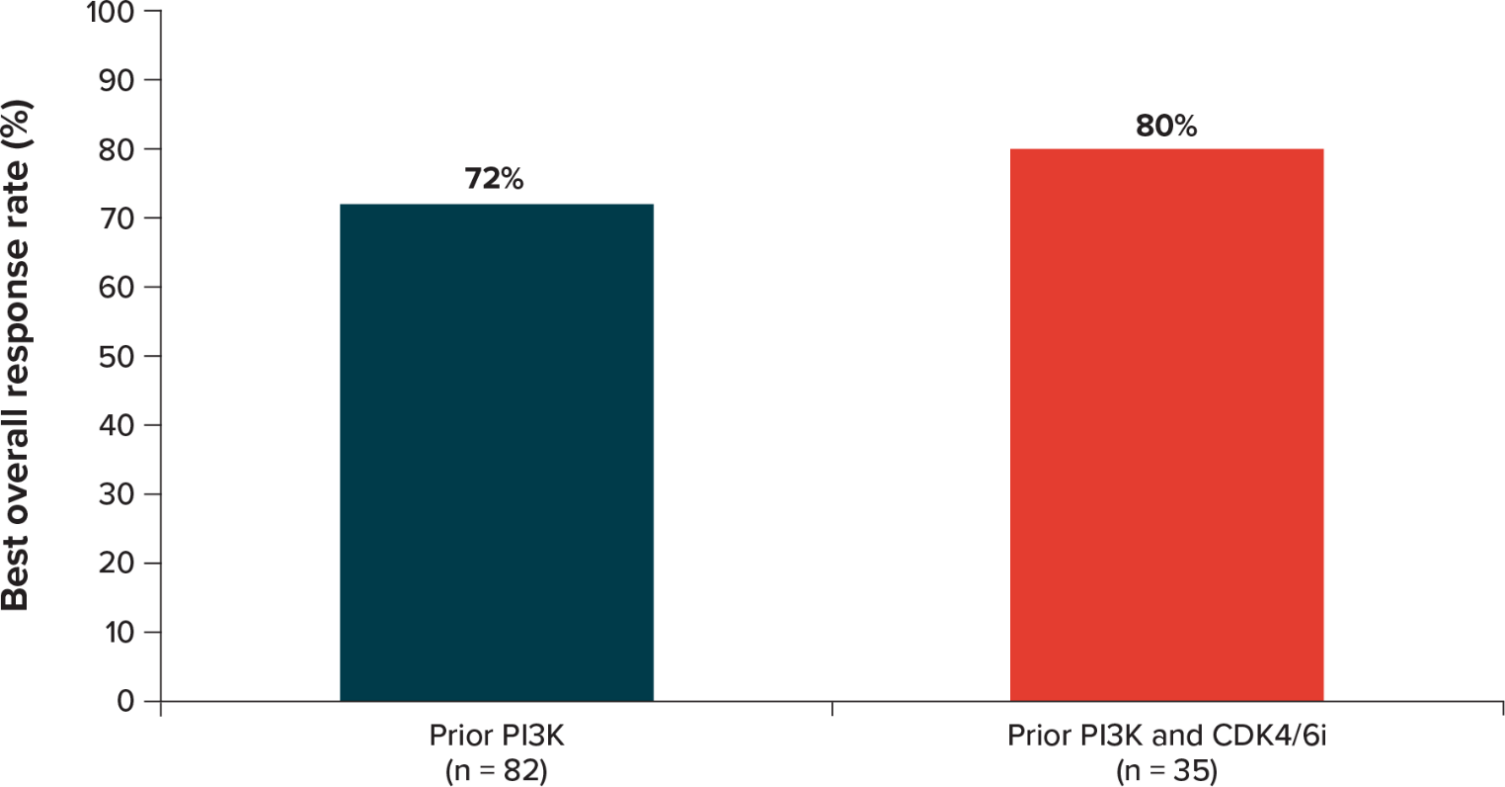
Physician-reported real-world best overall response to eribulin therapy. This figure represents patients who achieved physician-reported best overall response of CR or PR. CDK4/6i = CDK4/6i = cyclin-dependent kinases 4/6 inhibitors; CR = complete response; PI3K = phosphoinositide 3 kinase; PR = partial response

In the overall patient population (n = 82), according to physician-reported treatment response taken from patient medical charts, the real-world best overall response with eribulin treatment was reported to be CR (12.2%), PR (59.8%), SD (19.5%), and PD (7.3%) (Fig. 1). The real-world best overall response rate (CR + PR) with eribulin treatment was 72.0% (n = 59), and the clinical benefit rate (CR + PR + SD ≥ 24 weeks) was 74.4% (n = 61). Most of the treatment response assessments (56.1%) were based on the Response Evaluation Criteria in Solid Tumors (RECIST) guidelines [19] or the WHO (World Health Organization) criteria [20]. In addition, radiological assessments (computerized tomography scan, 53.7%; positron emission tomography scan, 24.4%) and other clinical and patient factors (patient symptoms, 35.4%; physical examination, 32.9%; patient physical performance, 23.2%) were reported to be used frequently in assessing clinical response. In patients who received both a prior PI3K inhibitor and a prior CDK4/6i (n = 35), physician-reported best overall response with eribulin treatment was CR (2.9%), PR (77.1%), SD (8.6%), and PD (11.4%).

**Fig. 2:**
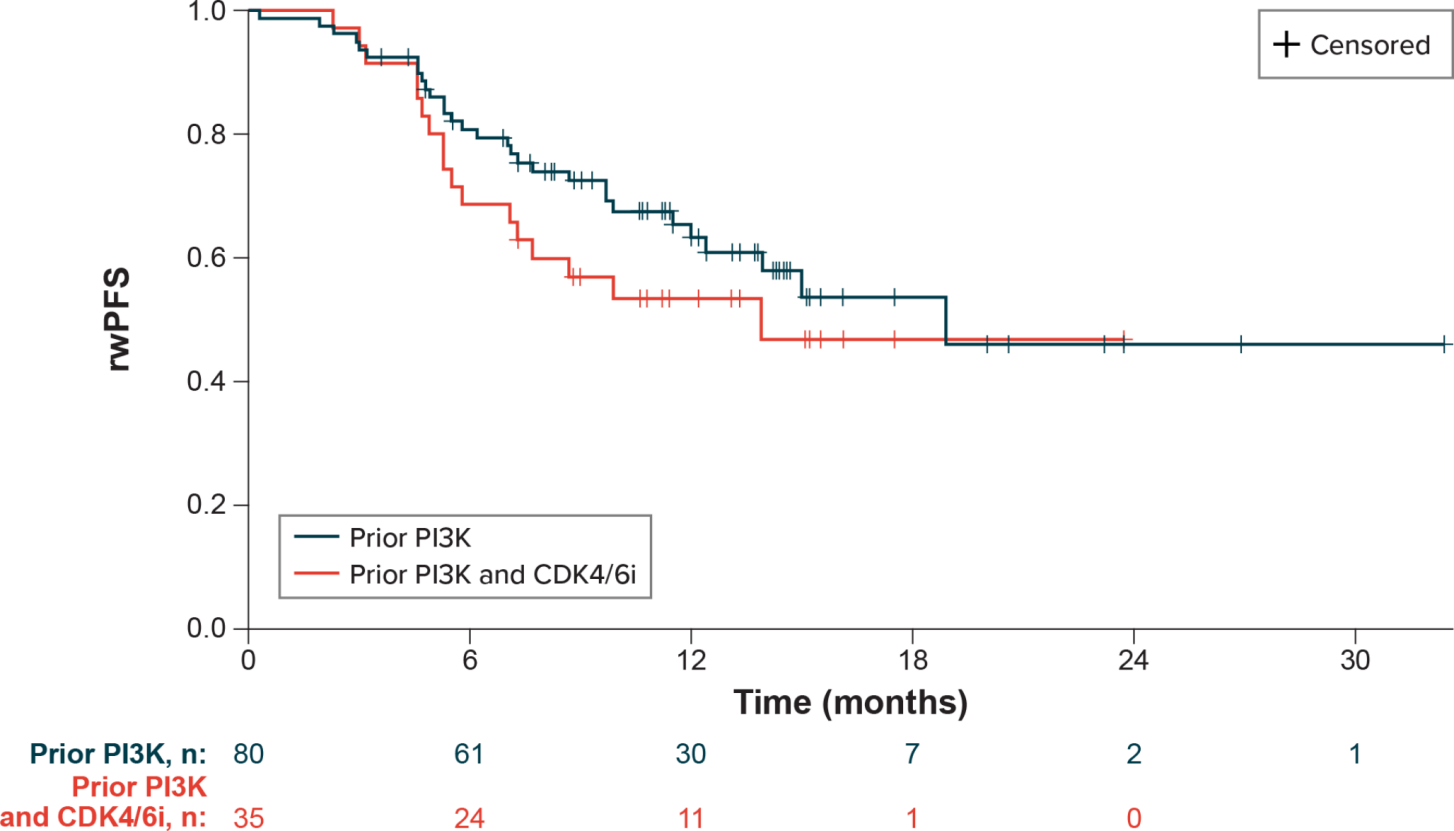
Real-world progression-free survival on eribulin following phosphoinositide 3-kinase inhibitor therapy. CDK4/6i = cyclin-dependent kinases 4/6 inhibitors; PI3K = phosphoinositide 3 kinase; rwPFS = real-world progression-free survival

From the date of eribulin therapy initiation, 37.5% of 80 patients experienced progression or death (2 patients with missing progression dates were excluded from this analysis). The median rwPFS was 18.9 months overall (95% CI, 12.4-not estimable [NE]), and the estimated rwPFS rates at 12 and 24 months were 63.3% (95% CI, 50.5-73.7%) and 46.1% (95% CI, 27.2-63.1%), respectively (Table 3; Fig. 2). The median rwPFS was not reached in patients who received eribulin as second-line therapy (n = 35); estimated rwPFS rates at 12 and 24 months for patients taking second-line eribulin were 69.9% (95% CI, 47.5-84.1%) and 51.6% (95% CI, 22.7-74.4%), respectively. The median rwPFS among those who received eribulin in the third or later line of therapy (n = 45) was 15 months (95% CI, 7.7–8.8); the 12-month rwPFS rate for patients with third-line or later eribulin use was 58.9% (95% CI, 42.8-71.8%). In the subgroup of patients treated with both prior PI3K inhibitor and prior CDK4/6i, the median rwPFS was 13.9 months (95% CI, 7.1-NE) and the estimated rwPFS rate at 12 months was 53.5% (95% CI, 35.6-68.5%).

**Table 3:** Real-World Progression-Free Survival and Overall Survival Following Initiation of Eribulin Therapy

	Patients with prior PI3K inhibitor treatment (n = 82)	Patients with prior PI3K inhibitor and CDK4/6i treatment (n = 35)
**rwPFS**		
Total patients	80	35
Patient with progression or death, n (%)	30 (37.5)	17 (48.6)
Patients censored, n (%)	50 (62.5)	18 (51.4)
rwPFS, median (95% CI), months	18.9 (12.4-NE)	13.9 (7.1- NE)
rwPFS rate, % (95% CI)		
6 months	80.8 (70.2–88.0)	68.6 (50.5–81.2)
12 months	63.3 (50.5–73.7)	53.5 (35.6–68.5)
18 months	53.8 (38.6–66.8)	46.8 (27.3–64.2)
24 months	46.1 (27.2–63.1)	--
**OS**		
Total patients	81	35
Patient with death event, n (%)	21 (25.9)	13 (37.1)
Patients censored, n (%)	60 (74.1)	22 (62.9)
OS, median (95% CI), months	NE (18.3-NE)	NE (12.2-NE)
OS rate, % (95% CI)		
6 months	91.4 (82.7–95.8)	85.7 (69.0-93.8)
12 months	82.6 (72.4–89.3)	70.9 (52.7–83.2)
18 months	67.9 (53.0–79.0)	53.9 (31.9–71.6)
24 months	63.9 (47.7–76.3)	--

CDK4/6i = cyclin-dependent kinases 4/6 inhibitors; CI = confidence interval; NE = not estimable; OS = overall survival; PI3K = phosphoinositide 3 kinase; rwPFS = real-world progression-free survival

NOTE: Patients with incomplete data on progression or death were excluded from the analyses of rwPFS (n = 2) and OS (n = 1).

**Fig. 3:**
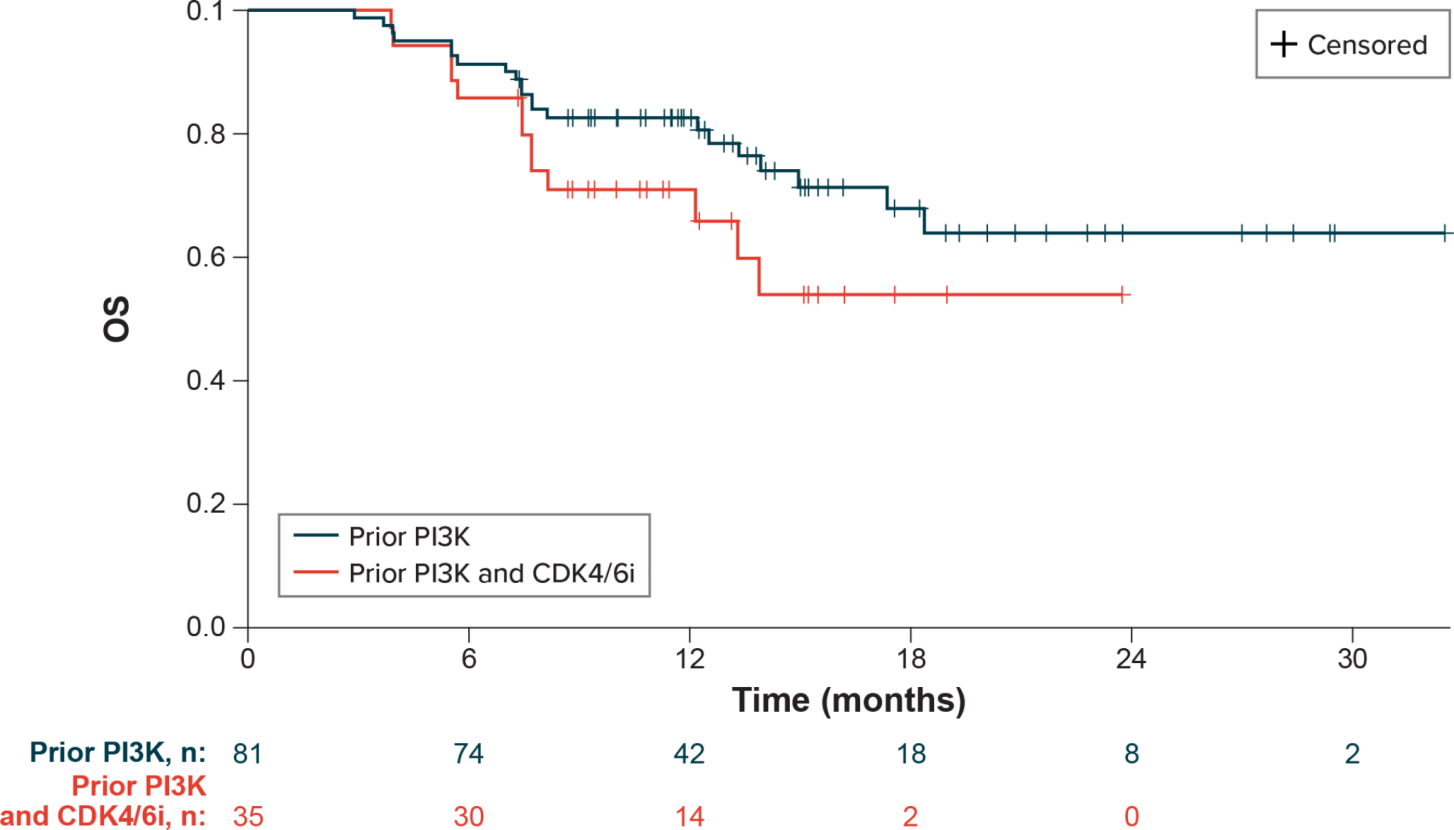
Overall survival on eribulin following phosphoinositide 3-kinase inhibitor therapy. CDK4/6i = cyclin-dependent kinases 4/6 inhibitors; OS = overall survival; PI3K = phosphoinositide 3 kinase

Median OS from eribulin initiation in the overall patient population was not reached; the estimated OS rates at 12 and 24 months were 82.6% (95% CI, 72.4-89.3%) and 63.9% (95% CI, 47.7-76.3%), respectively (Table 3; Fig. 3). The median OS was not reached for patients who received eribulin as second-line therapy (n = 37); estimated OS rates at 12 and 24 months for patients with second-line eribulin use were 91.9% (95% CI, 76.9-97.3%) and 81.5% (95% CI, 58.6-92.4%), respectively. The median OS among those who received eribulin in the third line or later (n = 44) was 18.3 months (95% CI, 13.3-NE); the 12-month OS rate for patients with third-line or later eribulin use was 74.7% (95% CI, 59.0-85.1%). In the subgroup of patients treated with both prior PI3K inhibitor and prior CDK4/6i, median OS was not reached; the estimated OS rate at 12 months from eribulin initiation was 70.9% (95% CI, 52.7-83.2%).

## Discussion

Our retrospective medical record review study is the first to evaluate the treatment patterns and clinical outcomes associated with eribulin therapy in patients with MBC who received prior treatment with a novel PI3K inhibitor in the US. The study population predominantly represented HR+/HER2– biomarker subtype, which is expected due to the approved indication of alpelisib. Patients were from all geographic regions of the US and were included regardless of race or ethnicity, physical performance status, or comorbidities.

This real-world study shows that most patients initiated eribulin in the second- (45%) or third-line (38%) setting after MBC diagnosis, and the estimated median time to eribulin discontinuation was slightly under 1 year. Our analyses showed that a majority, nearly three-quarters of the study sample, achieved physician-reported best overall response reported as complete or partial response during eribulin treatment, median rwPFS was 18.9 months, and median OS was not reached. In the subgroup of patients also treated with a prior CDK4/6i, 80% achieved complete or partial response with eribulin treatment, and median OS was not reached.

A recent systematic literature review of the real-world use of eribulin in the second line or later in MBC by Chabot et al. reported that the median rwPFS from the start of eribulin treatment ranged from 2.3 months to 14.7 months, while the median OS ranged from 6.9 months to 28.0 months [21]. In the current study, the median rwPFS from eribulin treatment initiation was estimated to be 18.9 months (12.4-NE); while the median OS in our study was not reached, the majority of patients (73%) were alive at the end of the study follow-up period. However, it should be noted that Chabot et al. [21] included some patients with triple-negative MBC, who typically have lower survival rates than patients with HR+/HER2– MBC [22, 23].

This initial evaluation of the treatment characteristics and clinical outcomes of eribulin following receipt of a PI3K inhibitor provides early real-world evidence in the evolving treatment landscape in MBC. Our study population was from all geographic regions of the US and from both academic and community practices. Patients who may be generally underrepresented in clinical trials were included in our study: 29.3% were non-White patients, and 11.0% had an ECOG performance status of 2 before eribulin initiation. This resulted in a diverse sample of patients more representative of the overall MBC patient population who received eribulin following receipt of a PI3K inhibitor and supports the generalizability of the effectiveness of eribulin in the broader real-world patient population.

The findings of this study should be viewed in the context of certain limitations. Patient data were collected via physicians who were willing to participate in the study; thus selection bias cannot be ruled out. However, we tried to minimize selection bias by selecting a broad sample of physicians and by guiding the physicians to randomly select eligible patients. In addition, differences are expected across participating physicians in terms of schedules and criteria used for the assessment of clinical endpoints (specifically treatment response and progression) in real-world clinical practice. And our findings are limited by the completeness and accuracy of the data captured by participating physicians in real-world clinical practice. Lastly, the size of the sample analyzed in this study was small and the follow-up duration was limited, owing to recent approval and availability of the PI3K inhibitor therapy. It is imperative that future research is undertaken to further explore treatment outcomes of eribulin with longer, mature follow-up data in patients previously treated with a PI3K inhibitor.

## Conclusion

This real-world study provides evidence on eribulin treatment characteristics and eribulin-related clinical outcomes among MBC patients previously treated with a PI3K inhibitor in US clinical practice. Nearly two-thirds of the study population were estimated to be alive after 2 years, with a median rwPFS of 18.9 months. Study findings suggest that eribulin may be considered as a potential treatment option for patients who failed a PI3K inhibitor in a prior therapy line for MBC.

## Data Availability

The data from this study is available upon reasonable request from Ravi Goyal at rgoyal@rti.org.

## Abbreviations

CDK4/6i: Cyclin-dependent kinases 4/6 inhibitors
CI: Confidence interval
CR: Complete response
ECOG: Eastern Cooperative Oncology Group
HER2+: HER2 positive
HER2–: HER2 negative
HER2: Human epithelial growth factor receptor 2
HR+: HR positive
HR: Hormone receptor
IRB: Institutional review board
MBC: Metastatic breast cancer
NE: Not estimable
OS: Overall survival
PD: Progressive disease
PI3K: Phosphoinositide 3 kinase
PR: Partial response
RECIST: Response Evaluation Criteria in Solid Tumors
rwPFS: Real-world progression-free survival
SD: Stable disease
US: United States
WHO: World Health Organization
